# Own price elasticities of the demand for sugar-sweetened beverages in Bangladesh

**DOI:** 10.1186/s12889-024-18544-4

**Published:** 2024-04-12

**Authors:** Rumana Huque, Abul Kalam Azad, Nasiruddin Ahmed

**Affiliations:** 1 Department of Economics, Social Science Building, University of Dhaka, Nilkhet, Dhaka-1000, Bangladesh; 2 ARK Foundation, Flat C3 and C4, House # 6; Road # 109 Gulshan 2, Dhaka, 1212 Bangladesh

**Keywords:** Sugar-sweetened beverages, Own price elasticities of the demand, The Deaton’s method, Bangladesh

## Abstract

**Background:**

Consumption of sugar-sweetened beverages (SSB) is a major global public health problem. Increasing the price of SSBs through taxation is an effective tool to reduce SSB consumption. Price-elasticity estimates are useful in measuring the effect of taxation on consumption. We estimated the own price elasticities of demand for SSBs in Bangladesh, which will inform how SSB taxes could affect behaviour.

**Methods:**

We used Household Income and Expenditure Survey (HIES) 2016 data, which is a nationally representative dataset at the household level across the country and is conducted using stratified random sampling method. Deaton’s method was used to estimate the price elasticities for SSBs in Bangladesh.

**Results:**

We found that the own price elasticity for SSBs varied between − 0.53% to -1.17% by types of SSBs in Bangladesh. The price elasticity for soft drinks was − 1.17, indicating that if the price of soft drinks increases by 10% via taxes, the quantity consumed of these beverages would reduce by 11.7%.

**Conclusion:**

This is the first study that estimates the own price elasticities of demand for SSBs in Bangladesh. Our results suggest to raise SSB prices through increased taxation in order to reduce SSB consumption and ensure public health gains in Bangladesh.

## Background

Liquids sweetened with various forms of added sugar are commonly known as sugar-sweetened beverages (SSB) [1]. Examples include regular soda, fruit drinks, sports and energy drinks, sweetened waters, pop, cola, tonic fruit punch, lemonade, sweetened powdered drinks, and coffee and tea beverages with added sugar [1, 2]. Consumption of SSBs is a major public health problem globally. There is ample evidence that soft drink intake is associated with increased obesity and increased health problems, such as, Type II Diabetes, kidney diseases, tooth decay, cardiovascular diseases and gout [3–5]. Keeping other things identical, if one consumes an SSB on a daily basis, s/he can gain weight by around 5 pounds in a year. One who drinks one to two cans daily possesses a 26% higher risk of developing type II diabetes mellitus with other metabolic syndromes, which can culminate in a premature death [6, 7]. SSB consumption is also found to be associated with adolescents’ poor mental health, including stress, depression, and suicidality [8, 9].

The consumption of SSBs in developing countries is increasing sharply, being highly induced by rapid urbanization and aggressive beverage marketing. The rate of SSB consumption is also concerning in Bangladesh, especially among children and youth. The Bangladesh Demographic and Health Survey 2022 reported that 32% of children aged 6–23 months were fed a sweet beverage in the last 48 h prior to the survey [10]. Surveys among school and university students suggest that 48% of the school children consumed soft drinks on a daily basis [11] while most of the university students (95.4%) consume SSBs, 53.6% reported more than twice a week [12]. Low price, taste (refreshing and good), extensive advertisement, availability, and peer influence remain the main reasons for consuming SSBs [11, 12]. Evidence suggests that the intake of SSBs in Bangladesh is higher than milk intake for women of reproductive age (20–49 years) and for males of active ages (20–69 years) at the national level [13]. It was also found that the consumption of SSB was higher among older adolescent boys (15–19 years) compared to younger adolescent boys; however, SSB consumption was reported lower among older girls compared to their younger counterparts [14]. Our recent study shows that the household expenditure on beverages and sugar-added drinks is around 2% of monthly household expenditure. However, the spending on beverages and sugar-added drinks is alarming due to the displacement of household expenditure for essential commodities, including food, clothing, housing, education, and energy [15].

During the late 1980s, sugary drinks, or SSBs, emerged in the market of Bangladesh, with only two to three companies operating [16]. However, several global and local branded soft drinks (both carbonated and non-carbonated) are now available in the market, and the market structure has become very competitive. The total volume of soft drinks sold in Bangladesh (both on-trade and off-trade) grew at an annual average rate of 6.9% during 2011–2018 [1]. However, the real prices of SSBs decreased during 2004– 2018, leading to increased affordability and consumption of SSBs over the period.

Several fiscal policies have been recommended by the World Health Organization (WHO) to reduce the consumption of SSBs. There is comprehensive evidence that increasing the price of SSB through taxation reduces SSB consumption, especially when the baseline consumption levels are high [17]. The effectiveness of a tax increase is also subject to the increase in the price of such products resulting from the tax change as well as the responsiveness of quantity demanded to a change in price (defined as the price elasticity of demand), among other factors [18]. Price-elasticity estimates are useful tools in measuring the effect of taxation on consumption.

Despite the growing consumption of SSBs, there is limited evidence on SSB taxation and the responsiveness of quantity demanded to a change in SSB price in Bangladesh. It is expected that SSB consumption will follow the basic demand law; that is, an increase in price will reduce consumption. However, a diverse range of SSB products are available in the market, and their price responsiveness may also vary. The price elasticity estimates can help the policymakers design appropriate taxation policies for different SSBs to reduce their consumption and avoid brand or product substitution. Price elasticities can also be used for tax modelling.

The SSBs that are domestically produced in Bangladesh are taxed by two general taxes: (a) Value-added tax (VAT) which is levied at a single rate of 15%; and (b) Supplementary duty (SD) for domestically produced SSBs which is 25% for the carbonated beverages and 35% for energy drinks [16]. SD on imported SSB is 150% [16]. Bangladesh can potentially make major public health gains by adopting appropriate taxation on SSBs. The objective of this study is to estimate the own price elasticities of demand for SSBs in Bangladesh, which will inform how SSB taxes could affect behaviour. The findings may contribute to the redesign and evaluation of current SSB taxation policies with the potential reduction of the prevalence of obesity, NCDs, and the associated premature deaths in Bangladesh. This study will also add to the limited evidence base of the own-price elasticity of demand for SSBs in low and middle-income countries.

## Data, variables and methodology

### Data and variables

We used Household Income and Expenditure Survey (HIES) 2016 data to estimate the own price elasticities of SSBs (soft drinks, horlicks, tea, and coffee) in Bangladesh. The HIES data is national-level data, and it is collected by the Bangladesh Bureau of Statistics (BBS) using the stratified random sampling method. The BBS is a government organization, which carries out the HIES survey at the household level in every five years and is also a major source of socio-economic information for the country. HIES 2016 is the latest round of data surveyed by the BBS. The detailed description of survey methods is outlined in the published report of BBS for HIES [19].

The SSBs can generally be defined as sugar-added drinks. Sugar-added drinks may encompass sugar-added soft drinks, carbonated drinks, sugary fruit drinks, sugary soda, and sweetened water [15, 20]. Allcott et al. (2019) emphasized that tea and coffee should be included in the definition of SSBs if additional sugar is added to tea and coffee [21]. Although Allcott et al. (2019) and Azad and Huque (2023) favor sugar-added tea and coffee being included in SSBs [15, 21], Zheng et al. (2015) preferred these to be considered as the substitute products of SSBs [22]. Following Allcott et al. (2019), as well as Azad and Huque (2023), we included tea and coffee in the definition of SSBs [15, 21]. Besides, most of the people in Bangladesh are used to consuming tea and coffee with added sugar [23].

The HIES 2016 does not explicitly define the SSB commodities during the data collection [15]. Therefore, we classified SSB commodities into five categories for our study based on the availability of the data. The five categories of SSB commodities contain soft drinks, horlicks, tea and coffee (in kilograms-kg), tea (in cups), and coffee (in cups). In HIES 2016, household spending on soft drinks is reported in kg, both the consumption inside and outside of households [19]. Therefore, we aggregated the inside and outside consumption of soft drinks. In the case of horlicks, spending for horlicks inside the household is reported as the expenditure for horlicks. As tea and coffee are bought in kg for consumption inside household, we considered the tea and coffee (in kg) as the dependent variable. On the other hand, since tea and coffee are consumed as ‘cups’ outside of the household, we treated these as separate dependent variables, i.e., tea (cups) and coffee (cups). These five categories of SSBs are considered as the dependent variables.

The socio-economic variables, including household yearly expenditure, household size, household head education, gender of the household head, the proportion of earners in the households, the proportion of adult members in the household, having refrigerators, and household migration status are included as the explanatory variables.

### Empirical model

Prices data, including the prices of SSBs, generally are not reported in the household survey data. However, one can estimate the price elasticity of demand without the data of price. In this circumstance, Deaton’s method is widely used to estimate the own price elasticities using the unit value as the proxy of price. Furthermore, the Deaton method of estimating price elasticities assumes that prices vary across clusters but not within clusters. The price variation across clusters means households living far apart (i.e., different villages or clusters) should experience different prices. On the other hand, households living near to one another (i.e., in the same village or purchase in the same market) should face the same price. The between-cluster variation of price is seen due to the significant transportation cost of moving goods from one place to another and other factors such as regional taxes or different rules in the same country. In contrast, factors like transportation cost and regional rules do not apply within the cluster (or in a village) and hence do not affect prices. We, therefore, used the Deaton method [24–26] to estimate the price elasticities for SSBs in Bangladesh. This method has been used to estimate the price elasticities of demand for tobacco products in several countries [27, 28] and SSBs in India [17] and Guatemala [29]. Deaton’s method uses both the quantity and quality of the goods in order to portray the representation of quality, quantity, and price information in the expenditure data for the commodities. Due to the lack of price information, unit values (UV) (expenditure/quantity) are used as the proxy for the prices of the commodities (i.e., UV = expenditure on SSB commodities/ purchased amount of quantity for the SSB commodities). Since unit values are not identical to actual price data, they have two potential problems- quality shading and measurement errors.

To explain the quality variations and measurement error, Deaton’s method uses the following two equations of unit value and budget share (following the framework of Deaton A. and John et al., 2019) [26, 27]:

1 $$\text{ ln}\,{UV}_{hc}={\delta }^{1}+{\theta }^{1}ln{z}_{hc}+{\phi }^{1}{H}_{hc}+\omega ln{\mu }_{c}+{\epsilon }_{hc}^{1}$$

2 $${w}_{hc}={\delta }^{0}+{\theta }^{0}ln{z}_{hc}+{\phi }^{0}{H}_{hc}+\sigma ln{\mu }_{c}+\left({f}_{c}+{\epsilon }_{hc}^{0}\right)$$

In the first equation, the unit value, the proxy of prices, is defined as the total household expenditure on Sugar-Sweetened Beverage (SSB) commodities divided by the quantity purchased for the commodity. $ln \,{UV}_{hc}$ is the natural logarithm of unit value for the household $h$ in cluster $c$, $ln{z}_{hc}$ indicates the log of household yearly expenditure, ${H}_{hc}$ indicates household related variables (such as household size, education and gender of the household head, the proportion of earners in the households, the proportion of adult members in the household, having refrigerators, and household migration status), $ln{\mu }_{c}$ is the unobserved prices, and ${\epsilon }_{hc}^{1}$ is the disturbance term that includes the usual unobservables and measurement error in unit values. On the other hand, the second equation of budget share tells us about the standard demand equation, where ${w}_{hc}$ is the expenditure share of the SSB commodities in the household total expenditure, and ${\epsilon }_{hc}^{0}$ is the additional error term with the cluster fixed effect, ${f}_{c}$. The disturbance term ${\epsilon }_{hc}^{0}$ also includes unobservable factors and measurement error in quantities. Therefore, the error term of the standard demand equation is correlated with the error term in the unit value equation.

Since $ln{\mu }_{c}$ is the unobserved price, Eqs. 1 and 2 are estimated excluding this unobserved price. As a result, excluding the unobserved price from Eqs. 1 and 2 will make the estimated parameter biased. Since any unobserved effect is contained in the error term, the unobservable prices are also part of the composite errors of Eqs. 1 and 2. It implies that the error terms of Eqs. 1 and 2 are correlated. This correlation is captured in their covariance term, ${\sigma }^{10}$. Therefore, to estimate the unbiased estimator of the parameters, we need to correct the potential biases from the estimators. Equation (6.1) and (4) address the problem of unobserved price in the estimation of the parameters $\omega$, and $\sigma$. Equation (4) included $\widehat{\gamma }$ that was estimated by including the covariance term, ${\sigma }^{10}$, of Eqs. 1 and 2, and Eq. (6.1) also addresses the biases by including $\widehat{\sigma }$ estimator. Therefore, the potential biases from unobserved prices are corrected in this way. The first equation of unit values also says that unit values do not depend on cluster fixed effect. Equation 1 of prices (unit value equation) does not include cluster-fixed effect, ${f}_{c}$ since Deaton (1997) showed that cluster-fixed effects are uncorrelated with price, though cluster-fixed effect is likely correlated with household characteristics [26]. Conditional on prices, unit values only depend on quality effects and measurement error. Moreover, the addition of the fixed effect in the budget share equation may eliminate the link between unit values and prices [26, 27]. Besides, Deaton assumed no price variation within the cluster. Thus prices are not necessary in the above regression.

Quality shading occurs when an increase in the price of a good does not cause a decrease in the quantity demanded. Rather, people may shift their purchases to lower-quality goods. The unit value equation tells us about the presence of quality effect if there is a significant positive relationship between unit value and household expenditure (a positive sign of ${\theta }^{1}$ in Eq. 1 above). After knowing the pattern of the quality shading effect in Eq. 1, one can correct this problem in the estimation of elasticity (Eq. 7) through Eq. 6.1 to 6.3. On the other hand, since unit values are calculated from the reported quantities and expenditure, measurement error contained in quantity may be transferred to measurement of unit value. As a result, the unit value may likely misrepresent the price, and, therefore, measurement error occurs [26]. The measurement error is generally corrected through the errors in variable regression (Eq. 5).

The aim of estimating elasticity requires estimating the equations of unit value and demand at the cluster level. The cluster-level average unit value and average demand equation can be represented after eliminating the household expenditure and household-level variables as

3 $${y}_{c}^{1}=\frac{1}{{n}^{+}}\sum\nolimits _{1}^{{n}^{+}}(\text{ln}{UV}_{hc}-{\theta }^{1}ln{z}_{hc}-{\phi }^{1}{H}_{hc})$$

4 $${y}_{c}^{0}=\frac{1}{n}\sum _{1}^{n}({w}_{hc}-ln{z}_{hc}-{\phi }^{0}{H}_{hc})$$

Where ${y}_{c}^{1}$is the cluster-level average unit value, and ${y}_{c}^{0}$ is the cluster-level average demand. In the above two equations, we remove the household subscript $h$ from the equation as we represent the equations at the cluster level. In addition, ${n}^{+}$ is number of households in the cluster that purchased positive amount of SSB commodities, while $n$ is the number of households in the cluster. Given the assumption of price variation in the between clusters but not within the cluster, the price elasticity of demand can only be estimated by observing how cluster level price changes affect the cluster-level demand. In order to do that, we need to regress the cluster average demand $\widehat{{y}_{c}^{0}}$ on average cluster unit value $\widehat{{y}_{c}^{1}}$ (regressing Eq. 4 on Eq. 1). Therefore, the coefficient of regressing average cluster demand on average cluster price tells us about the response of average cluster demand due to the change in average cluster unit values. The estimated regression gives us the estimated parameter, say $\widehat{\gamma }$, that works for the correction of measurement error in the unit value. The coefficient, $\widehat{\gamma }$, can also be obtained through the equation as follows:

5 $$\widehat{\gamma }=\frac{Cov \left({y}_{c}^{1}, {y}_{c}^{0}\right)-\frac{{\sigma }^{10}}{n}}{Var \left({y}_{c}^{1}\right)-\frac{{\sigma }^{11}}{{n}^{+}}}$$

Where ${\sigma }^{10}$ is the estimate for the covariance of the disturbance term in Eqs. 1 and 2, whereas ${\sigma }^{11}$ is the estimate for the variance of the disturbance term in Eq. 1. The coefficient, $\widehat{\gamma }$, gives the standard measurement to correct for measurement error. Equation 5 shows that the coefficient, $\widehat{\gamma }$, corrects the measurement error through the covariance and variance by taking the deviation from cluster level to household level for both covariance and variance. We also need the estimated coefficients of the unobserved price that were used in Eqs. 1 and 2 to estimate the price elasticities for SSB commodities. The coefficients for unobserved price can be obtained through the following equations:

6.1 $$\widehat{\omega }=1-\frac{{\widehat{\theta }}^{1}\left(\stackrel{-}{w}-\widehat{\sigma }\right)}{{\widehat{\theta }}^{0}+\stackrel{-}{w}}$$

6.2 $$\widehat{\sigma }=\frac{\widehat{\gamma }}{1+(\stackrel{-}{w}-\widehat{\gamma })\widehat{\eta }}$$

6.3 $$\widehat{\eta }=\frac{{\widehat{\theta }}^{1}}{{\widehat{\theta }}^{0}+\stackrel{-}{w}(1-{\widehat{\theta }}^{1})}$$

Where $\widehat{\omega }$ and $\widehat{\sigma }$ are the estimated coefficients from the equations of (1) and (2) for the representation of unobserved prices, and $\stackrel{-}{w}$ is the mean share of SSB commodities in total household expenditure. ${\widehat{\theta }}^{1}$ and ${\widehat{\theta }}^{0}$ are estimated coefficients of total household expenditure in Eq. 1 and respectively. $\widehat{\gamma }$ is the estimated coefficient for the regression of average cluster-level demand to average cluster-level unit value. Finally, according to Deaton’s method, the price elasticity of demand that also adjusts quality correction can be portrayed as follows:

7 $$\widehat{{e}_{p}}=\left(\frac{\widehat{\sigma } }{\stackrel{-}{w}}\right)-\widehat{\omega }$$

Where, $\widehat{{e}_{p}}$, estimates the price elasticity of SSB commodities using the estimated coefficients $\widehat{\omega }$, $\widehat{\sigma }$ and $\widehat{\eta }$. Using Eq. (7), we estimated the price elasticity of the five categories for SSB commodities, i.e., soft drinks, horlicks, tea and coffee (in kilograms-kg), tea (in cups), and coffee (in cups).

Model specification plays a very crucial role in determining the effectiveness of an econometric model for evidence-based policies. A correctly specified model yields the causal linkage between the outcome and policy variable. Model misspecification generally arises mostly due to the exclusion of important independent variables or adding some irrelevant variables. To test whether our chosen model is correctly specified, generally, the ‘Link Test’ [30, 31] and the ‘Ramsey RESET’ tests are used. We exploited both Link Test and Ramsey RESET to carry out as the test of model specification. Both tests are carried out under the null hypothesis that the model is correctly specified. The regression equation for the Link Test is as follows in Eq. 8:

8 $$y=\alpha +{\beta }_{1}\widehat{y}+{\beta }_{2}{\widehat{y}}^{2}+e$$

Where regression 8 is estimated after estimating the original equation $y=f\left(X\beta \right)$and, $y$is the dependent variable (the log of unit value, expenditure share). $X$ are all the independent variables used in Eqs. 1 and 2 (household yearly expenditure, household size, age, education and gender of the household head, the proportion of earners in the households, the proportion of adult members in the household, having refrigerators, and household migration status). $\widehat{y}$ is regression prediction, ${\widehat{y}}^{2}$ is the squared of regression prediction, and $e$ is the standard regression error term. The Link Test says that if model is correctly specified, we should not find any additional explanatory variables except by chance that are statistically significant [30, 31]. More specifically, we will not get statistically significant coefficient for $\widehat{y}$, especially for ${\widehat{y}}^{2}$.

On the other hand, Ramsey RESET test (Ramsey, 1969) is carried out is as follows [32]:

9 $${y}_{i}={x}_{i}\beta +{v}_{i}\rho +{\epsilon }_{i}$$

Where ${x}_{i}$ is the vector of independent variables, ${v}_{i}$ is $\widehat{{y}_{i}^{2}}, \widehat{{y}_{i}^{3}}, \widehat{{y}_{i}^{4}}$, and ${\epsilon }_{i}$is the regression disturbance term. The predicted $y$ is normalized to a value of 0–1. If the model is correctly identified, the F-test will not be statistically significant.

## Results

Table 1 presents summary data of the household consumption of different SSBs by their socio-economic status. It shows that those who consume horlicks had the highest monthly income (BDT 21,932), while the lowest monthly income is reported for the households that consume tea (outside). Similarly, the consumption pattern is also similar across the SSB consumption group. The greatest household size is reported for households who have tea and coffee consumption (kg) inside the households, and the lowest is found for the households who do not have any consumption for SSB commodities.

**Table 1:** Summary statistics of household basic indicators by different SSBs

Variable	Do not have any SSB consumption	Soft drink consumption	Horlicks consumption	Tea & coffee consumption (inside home)	Tea (cups) consumption	Coffee (cups) consumption
Household monthly income (BDT)	17632.21 (369,266)	19148.44 (66498.85)	21931.59 (35360.86)	18084.42 (66574.78)	14559.38 (57642.66)	16677.55 (22150.65)
Household monthly consumption (BDT)	10820.257 (12731.41)	20888.33 (16883.94)	23936.71 (16808.53)	19211.56 (15417.01)	15289.23 (12163.04)	18791.69 (17364.91)
Household size (number)	3.70 (1.53)	4.247 (1.57)	4.403 (1.60)	4.436 (1.68)	4.188 (1.52)	4.293 (1.62)
Proportion of adult members in the household	0.67 (0.22)	0.641 (0.21)	0.63 (0.21)	0.64 (0.21)	0.66 (0.21)	0.66 (0.21)
Proportion of earners in the household	0.32 (0.22)	0.32 (0.21)	0.29 (0.19)	0.29 (0.19)	0.334 (0.19)	0.327 (0.19)
Education of household head (years)	6.90 (3.50)	7.81 (3.88)	8.95 (4.23)	7.92 (3.98)	7.20 (3.73)	7.79 (3.96)
Gender of household head (= 1 if male)	0.81 (0.39)	0.86 (0.34)	0.82 (0.39)	0.84 (0.36)	0.93 (0.25)	0.93 (0.25)
Age of household head (years)	45.10 (14.928)	43.443(13.308)	44.12 (13.837)	45.27 (13.697)	44.44 (13.607)	44.57 (13.523)
Refrigerator (= 1 if having a refrigerator)	0.12 (0.321)	0.30 (0.459)	0.42 (0.494)	0.29 (0.455)	0.17 (0.378)	0.24 (0.425)
Migration (= 1 if at least a member migrated inside or outside of the country)	0.09 (0.282)	0.12 (0.323)	0.15 (0.357)	0.14 (0.344)	0.06 (0.246)	0.08 (0.266)
Health condition (= 1 if any member of a household was sick in the last twelve months)	0.45 (0.498)	0.52 (0.500)	0.56 (0.496)	0.59 (0.492)	0.49 (0.500)	0.52 (0.499)
No of observations	14,165	6,078	958	10,397	26,833	2,307

Statistics are mean values. Standard deviations are in the parenthesis

Although the proportion of adult members is found to be largest among the households who did not consume SSB, the largest proportion of earners in the households was found in households that have an expenditure on tea and coffee outside of the households (0.334 and 0. 327 respectively). In terms of obtaining education, the household heads belonging to the horlicks consumption are found to acquire the highest years of education (8.94 years). Table 1 also shows that most of the households are male-dominated regardless of the SSB consumption. The households who consume horlicks and, tea and coffee are reported to have greater members who migrated outside the households. The households who do not have consumption of sugary drinks faced a lower level of sickness among their members compared to the other households having the consumption of different types of SSBs.

We estimated the Analysis of Variance (ANOVA) to test whether the unit values vary across the clusters or geographical unit. Table 2 presents that the results of ANOVA analysis. It shows that significant F-statistics reject the null hypothesis of no variation of unit values across the clusters, as the Table 2 also shows that the F-values for all SSB commodities are statistically significant. Besides, the R-squared value indicates that cluster dummies describe more than half of the total variation of unit values (between 51 and 80%). The estimated results indicate at least 51% of the variation in unit values is explained by the between-cluster effects, which clearly hints that there are price variations across the clusters.

**Table 2:** Testing spatial variation in log unit values (${{H}}_{0}:$ There is no price variation between the clusters)

Type of SSB Goods	F-statistic	*p*-value	R-squared	n
Soft Drinks	4.890	0.000	0.575	6078
Horlicks	3.220	0.000	0.798	958
Tea and Coffee (Inside house consumption) in kg	11.790	0.000	0.608	10,397
Tea in cup (Outside house)	11.740	0.000	0.512	26,833
Coffee in cup (Outside house)	1.730	0.000	0.611	2307

Authors’ own calculation

The results obtained from regression 1 (Eq. 1) are presented in Table 3. Table 3 contains the estimated results from all of the SSB commodities. Estimated results from the natural logarithm of the household expenditure coefficient show the quality elasticity. The lower coefficient value indicates the lower quality shading in the unit value. Table 3 shows that quality shading in soft drinks and horlicks is low and insignificant.

On the other hand, the remaining three categories of SSBs portray quality elasticities between 2 and 13%. Although these are statistically significant coefficients, the magnitudes of elasticity are small. However, the results indicate that there is quality shading in unit values for most of the SSBs (tea & coffee (inside household consumption in kg), tea (cups), and coffee (cups)).

**Table 3:** Estimated results from the regression of (log) unit value

Explanatory variables	Soft Drinks	Horlicks	Tea and Coffee (Inside house consumption) in KG	Tea Cup (Outside house)	Coffee Cup (Outside house)
Natural logarithm of household total yearly expenditure	0.003 (0.029)	0.111 (0.097)	0.038*** (0.013)	0.015*** (0.005)	0.138** (0.065)
Household size	0.006 (0.009)	-0.058** (0.027)	-0.006* (0.004)	-0.002 (0.002)	-0.005 (0.021)
Proportion of adult members in the household	0.038 (0.065)	-0.326 (0.218)	-0.049* (0.029)	-0.009 (0.011)	-0.057 (0.165)
Proportion of earners in the household	-0.032 (0.068)	0 0.074 (0.251)	0.027 (0.032)	-0.002 (0.012)	0.295* (0.170)
education of household head in years	-0.005 (0.003)	-0.005 (0.011)	-0.000 (0.001)	0.000 (0.000)	0.012 (0.008)
Gender of household head	0.034 (0.039)	0.029 (0.118)	0.018 (0.016)	0.006 (0.008)	-0.225** (0.109)
Age of household head in years	-0.002 (0.001)*	0.000 (0.003)	-0.001 (0.000)	-0.000 (0.000)	0.001 (0.002)
Refrigerator; =1 if having a refrigerator and 0 otherwise	0.026 (0.028)	0.065 (0.101)	0.013 (0.013)	-0.013** (0.005)	0.129* (0.075)
Migration; =1 if at least a member migrated inside or outside of the country and = 0 otherwise	0.005 (0.038)	0.114 (0.116)	0.033** (0.016)	-0.016** (0.008)	-0.267** (0.110)
Constant	4.187*** (0.330)	4.986*** (1.128)	5.435*** (0.145)	1.307*** (0.054)	-0.011 (0.761)
No of households	4095	723	7031	16,088	1,454
R-squared	0.639	0.832	0.628	0.543	0.683

*p < 0.10, **p < 0.05, ***p < 0.01; Standard errors are in parenthesis

Household size is negatively associated with the unit values for the horlicks and tea and coffee (in KG), though the coefficients are not significant for other cases. On the other hand, the proportion of adult members is negatively associated with unit values for tea & coffee (KG), while positively associated with Coffee (cups). The migration status of a household positively affects the unit value of inside tea & coffee and negatively for tea and coffee outside the household.

On the other hand, estimated results from the budget share equation (Eq. 2) are reported in Table 4. The obtained results show that the coefficients of the budget share equation for household total expenditure negatively affect the budget shares of all SSB commodities except horlicks. The results are statistically significant at 1% level of significance. Although some other explanatory variables significantly affect the budget share of SSB commodities in the household total expenditure, most of the magnitudes are very small. Besides, the level of significance for PSU (primary sampling units) for all model is also statistically significant (Table 4).

**Table 4:** Estimated results from the regression of budget share

Explanatory variables	Soft Drinks	Horlicks	Tea and Coffee (Inside house consumption) in KG	Tea Cup (Outside house)	Coffee Cup (Outside house)
Natural logarithm of household total yearly expenditure	-0.002*** (0.000)	0.002 (0.002)	-0.003***(0 0.000)	-0.009*** (0.000)	-0.001*** (0.000)
Household size	0.000 **(0.000)	-0.001* (0.000)	0.000 (0.000)	-0.000*** (0.000)	0.000 (0.000)
Proportion of adult members in the household	0.002** (0.001)	-0.003 (0.000)	0.000 (0.000)	0.003*** (0.001)	0.000 (0.001)
Proportion of earners in the household	-0.000 (0.001)	0.000 (0.003)	-0.000 (0.000)	0.004*** (0.001)	0.001* (0.001)
Education of household head in years	0.000 (0.000)	-0.000 (0.000)	0.000 (0.000)	-0.000*** (0.000)	-0.000 (0.000)
Gender of household head	-0 0.001 (0.000)	0.003 (0.002)	0.000 (0.000)	0.005*** (0.000)	-0.000 (0.000)
Age of household head in years	-0.000*** (0 0.000)	-0.000 (0.000)	0.000*** (0.000)	-0.000 (0.000)	0.000 (0.000)
Refrigerator; =1 if having a refrigerator and 0 otherwise	-0 0.001* (0.000)	0.000 (0.002)	-0.000 (0.000)	-0.001*** (0.000)	0.000 (0.000)
Migration; =1 if at least a member migrated inside or outside of the country and = 0 otherwise	-0.000 (0.000)	0.000 (0.002)	0.000 (0.000)	-0.001*** (0.000)	0.000 (0.000)
Constant	0.037*** (0.004)	-0.007 (0.018)	0.043*** (0.002)	0.112*** (0.003)	0.017 (0.003)
PSU (primary sampling unit) F-value	3.726*** (0.000)	2.182*** (0.000)	8.030*** (0.000)	4.7333*** (0.000)	1.171*** (0.000)
No of households	4095	723	7031	16,092	1455
R-squared	0.613	0.783	0.630	0.492	0.619

*p < 0.10, **p < 0.05, ***p < 0.01; Standard errors are in parenthesis

Table 5 presents the own-price elasticities of demand for SSBs (soft drinks, horlicks, tea & coffee (kg), tea (cups), and coffee (cups)). All price elasticity coefficients are significant at 1% level of significance, except the price elasticity of coffee. Therefore, the elasticity varies between − 0.53% to -1.17%. Among the SSB drinks, soft drink is price elastic, while the other beverages and sugar-added drinks are inelastic commodities. Estimated results from Table 5 show that a 10% increase in the price of soft drinks reduces the quantity demand for soft drinks by about 11.7%, holding the socio-economic variables constant. It implies that soft drink is an elastic good in Bangladesh.

**Table 5:** Estimates of own-price elasticities of demand for various sugar-sweetened beverages (SSBs) in Bangladesh

Type of SSB Goods	Own Price Elasticity	[95% Conf. Interval]
Soft Drinks	-1.168*** (0.046)	[-1.258, -1.077]
Horlicks	-0.527*** (0.132)	[-0.785, -0.270]
Tea and Coffee (Inside house consumption) in KG	-0.822*** (0.054)	[-0.928, -0.715]
Tea Cup (Outside house)	-0.747*** (0.073)	[-0.889, 0.604]
Coffee Cup (Outside house)	-0.384 (7.942)	[-15.949, 15.182]

***p < 0.01; Bootstrap standard errors are in parenthesis. 95% CI are also obtained through the bootstrap method

On the other hand, the lowest magnitude of elasticity belongs to coffee, though the coefficient is not statistically significant. The elasticity of horlicks tells that a 1% increase in the price of horlicks reduces the consumption of horlicks by about 0.53% remaining the other variables constant. It implies that an increase in horlicks price reduces the least amount of horlicks consumption compared to soft drinks, tea and coffee (kg), and tea (cups) in Bangladesh. The elasticity of tea and coffee (kg) in household consumption is about − 0.82%. It says that a 1% increase in the price of tea and coffee (kg) reduces the quantity demand for tea and coffee (kg) by about 0.82%, holding all other variables constant. On the other hand, a 1% increase in outside tea (cups) consumption significantly decreases it consumption by about 0.75%.

Table 6 contains the major components that are required to estimate the price elasticities of the sugar-sweetened beverages (SSB) commodities reported in Table 5 above. More precisely, the coefficient values of Eqs. 5, 6.1, 4, and 6.3 with standard errors and 95% confidence interval are presented in the following Table 6. Although these components do not have any specific meanings, these are used to derive the price elasticities of demand for SSB commodities in the study.

**Table 6:** Major components required for the estimation of elasticity

Type of SSB Goods	$\boldsymbol{\widehat{\gamma}}$		$\boldsymbol{\widehat{\omega}}$		$\boldsymbol{\widehat{\sigma}}$		$\boldsymbol{\widehat{\eta}}$	
	Coefficient	95% CI	Coefficient	95% CI	Coefficient	95% CI	Coefficient	95% CI
Soft Drinks	-0.001 (0.000)***	[-0.002, -0.001]	0.995 (0.000)***	[0.994, 0.996]	-0.001 (0.000)***	[-0.002, -0.001	0.527 (0.656)	[-0.759, 1.812]
Horlicks	0.002 (0.001)***	0.001, 0.004]	0.950 (0.012)***	[0.925, 0.975]	0.002 (0.001)***	[0.001, 0.004]	17.461 (0.640)***	[16.207, 18.715]
Tea and Coffee (Inside house consumption) in KG	0.001 (0.000)***	[0.000, 0.002]	0.942 (0.004)***	[0.934, 0.949]	0.001 (0.000)**	[0.000, 0.002]	9.835 (0.427)***	[8.997, 10.673]
Tea Cup (Outside house)	0.004 (0.001)***	[0.002, 0.007]	0.979 (0.002)***	[0.975, 0.982]	0.004 (0.001)***	[0.002, 0.007]	1.570 (0.196)***	[1.186, 1.953]
Coffee Cup (Outside house)	0.002 (0.066)	[-0.127, 0.130]	0.871 (2.671)	[-4.365, 6.107]	0.001 (0.029)	[-0.055, 0.058]	123.950 (1.489)***	[121.032, 126.868]

**p < 0.05, ***p < 0.01; Bootstrap standard errors are in parenthesis. 95% CI stands for 95% Confidence Interval. 95% CI are also obtained through the bootstrap method

Table 7 shows the results of Link Test and Ramsey RESET test for testing omitted variables in the model. The table depicts that almost all of the coefficients from both tests are found statistically insignificant except for the expenditure share for tea cup (outside house), tea and coffee consumption (inside house), and tea-cup both for inside and outside house. Therefore, overall, it implies that the assumed econometric model is correctly specified. It’s not necessary to add or subtract any additional independent variables.

**Table 7:** Model specification using Link Test and Ramsey RESET test

Dependent Variable	Link Test			Ramsey RESET Test
	Constant	Regression prediction (y_hat)	Squared of regression prediction (y_hat^2^)	F-statistics (*p*-value)
	Expenditure share equation (Eq. 1)			
Log of unit value for Soft Drinks	150.822 (166.459)	-71.044 (79.511)	8.603 (9.495)	0.43 (0.735)
Log of unit value for Horlicks	− 25.708 (52.304)	9.583 (17.453)	-0.716 (1.456)	1.96 (0.119)
Log of unit value for Tea & Coffee (Inside house Consumption)	-100.898 (130.931)	35.513 (44.785)	-2.951 (3.830)	2.35 (0.070)*
Log of unit value for Tea Cup (Outside house)	-22.197 (29.327)	31.400 (40.162)	-10.408 (13.750)	20.80 (0.000)***
Log of unit value for Coffee cup (outside house)	0.592 (2.054)	1.704 (2.423)	-0.207 (0.711)	2.21 (0.086)*
	Expenditure share equation (Eq. 2)			
Expenditure share for Soft Drinks	0.001 (0.002)	0.588 (0.489)	29.357 (34.189)	0.96 (0.410)
Expenditure share for Horlicks	0.006 (0.006)	-1.487 (2.000)	224.617 (177.002)	3.05 (0.028) **
Expenditure share for Tea & Coffee (Inside house)	0.001 (0.001)	0.700 (0.170) ***	24.373 (13.285) *	0.29 (0.830)
Expenditure share for Tea Cup (Outside house)	0.004 (0.001) ***	0.459 (0.064) ***	17.674 (1.994) ***	10.30 (0.000) ***
Expenditure share for Coffee Cup (Outside house)	0.001 (0.001)	0.507 (0.544)	103.401 (108.763)	1.00 (0.391)

*p < 0.10, **p < 0.05, ***p < 0.01; Standard errors are in parenthesis for Link Test. However, probabilities are in parenthesis for Ramsey RESET test

## Discussion

This study, for the first time, estimates the own price elasticities of the demand for different types of SSBs in Bangladesh. We found that the own price elasticity for SSBs varied between − 0.53% to -1.17% by types of SSBs in Bangladesh. A recent study in India estimated the own-price elasticity of aerated or sugar-sweetened beverages (ASBs) as − 0.94 in the overall sample, which varied between − 1.04 and − 0.83 from low- to high-income households [17]. In Ecuador and Argentina, the own-price elasticities for SSBs varied between − 1.17 and − 1.33 [33], and − 1.10 and − 1.15 [34] respectively, depending on the household income quintile.

Our estimation of the price elasticity for soft drinks was − 1.17. This implies that if the price of soft drinks increases by 10% via taxes, the quantity consumed of these beverages would reduce by 11.7%. This is consistent with a number of previous studies. For example, the price elasticity for soft drinks was found − 1.06 in Mexico [35] and − 1.37 in Chile [36]. The greater price sensitivity among people consuming soft drinks indicates that the tax-induced price increase would generate larger public health gains to people.

The findings of our study indicate that an increase in prices of horlicks would reduce the horlicks consumption by the least amount compared to other SSBs in Bangladesh. As horlicks is generally consumed as child food and also during illness as energy-booster in Bangladesh, this might be a reason for the lowest response of horlicks consumption to its price rise.

The continuous growth in SSB consumption can be harmful for public health. The availability of SSBs in affordable price will encourage uptake among children, youth and the poor, resulting in larger adverse health consequences on future generations, especially poorer and marginalized population. Raising price through imposing appropriate SSB taxation can be an effective tool for reducing SSB consumption in Bangladesh, thereby decreasing obesity and health care expenditure [15, 37]. However, like many other countries, the current SSB taxation in Bangladesh is low, and the true costs of SSBs arising from public healthcare expenditures and other societal costs from excessive intake are not reflected in current market prices for SSBs [38]. There also remain tax differences between domestically produced and imported soft and energy drinks, and across molt beverages, fruit juices and flavored sweetened milks, which allows consumers to switch to low-price products as close substitutes in Bangladesh. SSBs are available in multiple-sized pack or bottle, making it affordable to consumers. All these factors might undermine the expected effects of SSB taxation in the country. Findings in our study suggest that the demand for SSBs is responsive to price changes. Hence, a considerable increase in the prices of SSBs through taxation will result in a marked reduction in SSB consumption and will also increase government revenue. However, the effectiveness of SSB taxation will largely depend on the appropriate tax structure and proper implementation. The tax structure should be designed to limit the scope of switching to lower-priced varieties of SSBs, and the tax must be annually adjusted for inflation and income growth to be consistent with the real value of SSB prices. A complex tax structure with multiple tax rates may also pose administrative challenges for revenue generation due to widespread tax avoidance among producers and consumers, resulting in limited effect of tax increases on reducing SSB consumption. A standard classification of sugary drinks based on sugar content and other ingredients can be defined [16]. Along with high SSB taxation, non-price measures including mass campaign, awareness raising, and innovative interventions need to be designed and implemented to reduce the information asymmetry [1, 35], thereby decreasing the SSB consumption in Bangladesh.

This study had a number of limitations. As the HIES 2021 data is not publicly available yet, we analysed the HIES 2016 data. The consumption pattern of SSB and related variables might have changed since 2016. Moreover, the self-reported data on quantities and expenditures at the household level were used for estimating the own price elasticities of SSBs. However, a number of studies used household-level data to estimate price elasticity of demand for SSBs and argued that as these products are usually consumed by most members of a household, a household can be considered as the basic unit of analysis. In addition, in the absence of an operational definition of SSB in the HIES 2016 dataset and limitations of the available data, we have categorized the SSB in line with the questions asked in the HIES 2016 survey and their classification of SSBs. Hence, some SSBs included in our study (e.g., tea and coffee) may not have added sugar in it. Despite the limitations, this study, for the first time, estimates the own price elasticities of the demand for SSBs in Bangladesh. Similar to many other countries, SSB needs to be defined properly and the classification in the data sets should distinguish between drinks with and without added sugar. This is also important for designing taxation on diverse types of SSB products. Further study is required to estimate the cross-price elasticities to show different substitutes and complements of SSBs. Moreover, own price elasticity by income groups and location (urban vs. rural) would help the policy makers to design and implement innovative interventions to curb SSB consumption.

## Conclusion

The increasing pattern of consuming SSBs is negatively affecting the healthcare system in Bangladesh. This increasing pattern is alarming as the country needs to reallocate its scarce resources from providing the essential health care to the uninvited diseases that arise due to the consumption of sugary drinks. The burden on the healthcare system emphasizes the necessity of reducing the consumption of SSBs. However, in order to formulate appropriate policies, policymakers need to know how the SSB consumption is responsive to the policy tools (like taxation). Therefore, we estimated the own price elasticities of different SSB commodities using national-level data. Our results confirmed that the demand for SSB commodities is significantly responsive to their prices.

This is the first study that estimates the own price elasticities of demand for SSBs in Bangladesh. Our results suggest to raise SSB prices through increased taxation in order to reduce SSB consumption and ensure public health gains in Bangladesh. SSB control needs to be prioritised in the government’s policy and practice, and a multisectoral approach is required to reduce SSB consumption.

## Data Availability

The datasets used and/or analyzed during the current study are available from the Household Income and Expenditure Survey, Bangladesh- [email: dg@bbs. gov.bd; web link: http://www.bbs.gov.bd/] on reasonable request.
